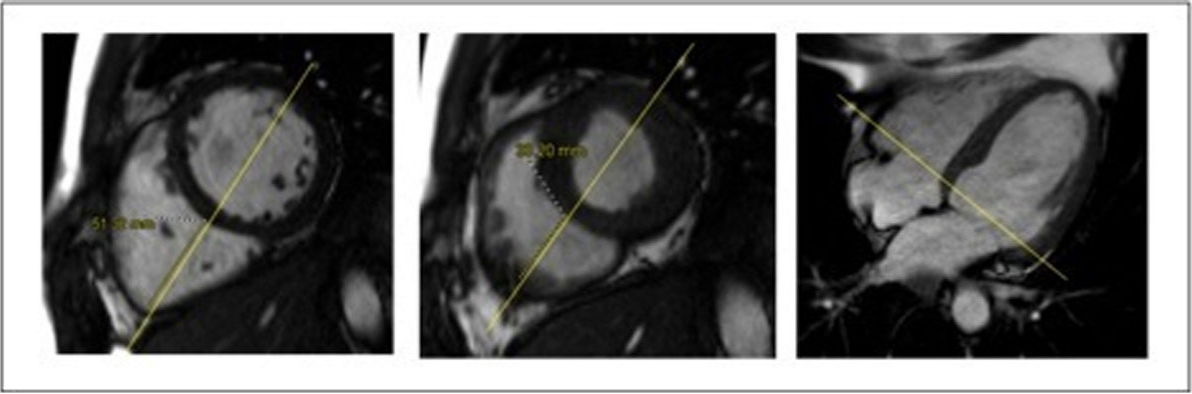# Accuracy of a new method for semi-quantitative assessment of right ventricular ejection fraction by cardiac MRI: right ventricular fractional diameter changes

**DOI:** 10.1186/1532-429X-15-S1-E80

**Published:** 2013-01-30

**Authors:** Emmanuelle Vermes, Nicolas Rebotier, Olivier Genee, Julien Pucheux, Anne Delhommais, Daniel Alison

**Affiliations:** 1 University Hospital of Tours, Chambray les Tours, France

## Background

Cardiovascular magnetic resonance (CMR) is the gold standard to measure right ventricular ejection fraction (RVEF). Longitudinal shortening is historically known to be the predominant part of its global systolic function and less attention has been paid to the transversal contraction. The aim of this study was to evaluate RV transverse motion in a large cohort of patients referred for CMR and assess its relationship with RVEF.

## Methods

We retrospectively analyzed the CMR scans of 300 consecutive patients referred for CMR between January and December 2010. Reference RV ejection fraction was determined from short axis sequences using the volumetric method. Transverse parameters called RV fractional diameter changes (FDC) were calculated after measuring RV diastolic (D) and systolic (S) diameters at basal and medium level in short axis view (respectively FBDC and FMDC) FBDC= 100 x (D-S)/D (Figure: FBDC=100 x (51.32 - 33.20) / 51.32 = 35.7%).We also measured the tricuspid annular plane systolic excursion (TAPSE) in the four chambers view as a longitudinal reference.

## Results

Population was divided into 2 groups according RVEF, 250 patients had a preserved RVEF (>40%) and 50 had a RV dysfunction (RVEF ≤40%). Both transverse and longitudinal motions were significantly reduced in the group with RV dysfunction (p<.0001). After ROC analysis, areas under the curve for FBDC ,FMDC and TAPSE, were respectively 0.79, 0.82 and 0.72 with the highest sensitivity and specificity of 68% and 88% for FMDC ( threshold at 19.9%) to predict RVEF. FMDC had an excellent negative predictive value of 93%.

## Conclusions

Right ventricular fractional diameter changes, especially at the medium level, appear to be accurate for semi quantitative assessment of RV function by CMR.

## Funding

No funding.

**Figure 1:**